# Immunomodulation in Administration of rAAV: Preclinical and Clinical Adjuvant Pharmacotherapies

**DOI:** 10.3389/fimmu.2021.658038

**Published:** 2021-04-01

**Authors:** Wing Sum Chu, Joanne Ng

**Affiliations:** ^1^Pharmacy Department, The Royal Marsden NHS Foundation Trust, London, United Kingdom; ^2^Gene Transfer Technology Group, Department of Maternal and Fetal Medicine, EGA Institute for Women’s Health, University College London, London, United Kingdom

**Keywords:** immunomodulation, immunosuppression, immune response, gene therapy, adeno associated virus, pharmacotherapies

## Abstract

Recombinant adeno-associated virus (rAAV) has attracted a significant research focus for delivering genetic therapies to target cells. This non-enveloped virus has been trialed in many clinical-stage therapeutic strategies but important obstacle in clinical translation is the activation of both innate and adaptive immune response to the protein capsid, vector genome and transgene product. In addition, the normal population has pre-existing neutralizing antibodies against wild-type AAV, and cross-reactivity is observed between different rAAV serotypes. While extent of response can be influenced by dosing, administration route and target organ(s), these pose concerns over reduction or complete loss of efficacy, options for re-administration, and other unwanted immunological sequalae such as local tissue damage. To reduce said immunological risks, patients are excluded if they harbor anti-AAV antibodies or have received gene therapy previously. Studies have incorporated immunomodulating or suppressive regimens to block cellular and humoral immune responses such as systemic corticosteroids pre- and post-administration of Luxturna^®^ and Zolgensma^®^, the two rAAV products with licensed regulatory approval in Europe and the United States. In this review, we will introduce the current pharmacological strategies to immunosuppress or immunomodulate the host immune response to rAAV gene therapy.

## Introduction

Adeno-associated virus (AAV) is a 26nm, non-enveloped virus of *Parvoviridae* family. It is 4.7kb single-stranded DNA genome containing 4 open reading frames (ORFs) (rep, cap, aap, and MAAP) flanked by inverted terminal repeats (ITRs) (1, 2). In therapeutic gene delivery, the viral ORFs are replaced by the desired transgene expression cassette and referred as recombinant AAV (rAAV). It has emerged as a leading vector to deliver genetic therapies due to its ability to transduce diverse cell types and safety profile.

A significant obstacle in clinical delivery of rAAV is host immune response triggered by rAAV capsid, genome, and therapeutic protein produced (3). Although AAV infection is non-pathogenic in humans, initial exposure induces humoral and cellular anti-capsid response that are reactive to rAAV due to capsid similarity (4, 5). Pre-existing neutralizing antibody (NAb) can effectively block rAAV transduction even at low levels (1:5) (6). Most rAAV clinical trials exclude seropositive patients; given the high seroprevalence (60% for AAV2), limiting patients suitable for rAAV therapy (7, 8). Furthermore *ex vivo* studies have shown predominantly pre-existing memory phenotype cytotoxic T lymphocytes (CTL), following exposure to rAAV can undergo expansion and potentially lead to elimination of transduced cells (9, 10).

After rAAV administration, capsid-derived epitopes can be presented by professional antigen presenting cells (APC) *via* major histocompatibility complex (MHC) class I pathway and activate CTL (11). The activation of CTL can result in targeted destruction of transduced cells, as observed in rAAV2 hemophilia B clinical trial (12). Despite initial stable therapeutic factor IX (FIX) expression (>10% activity) for 4 weeks, FIX levels gradually declined to baseline (<1%). This was associated with asymptomatic, self-limiting transaminitis, and corresponding changes in capsid-specific CTL population (5). In the subsequent study using AAV8, administration of steroids was able to negate this response and maintain therapeutic FIX levels albeit a 50-70% decline from peak levels (13). Moreover, transgene protein product-specific CTL was observed in human rAAV trials for Duchenne’s Muscular Dystrophy (14) and α-1-antitrypsin (15). Regulatory T cells (Treg) modulate immune tolerance towards transgene product and capsid that are vital to durable expression of therapeutic protein (16, 17). Although the full clinical significance of innate response to rAAV is unclear (18), unmethylated CpG motifs in rAAV vector genome interact with toll-like receptor (TLR) 9 present in plasmacytoid dendritic cells and Kupffer cells, releasing type I interferons activating cellular and humoral responses in mouse models (19, 20), and has been suggested as the cause of loss of expression in a rAAV8 hemophilia B trial (21). Furthermore, rAAV capsid-targeting TLR2, various DNA sensors, and complement activation may also play a role (22).

**Figure 1:**
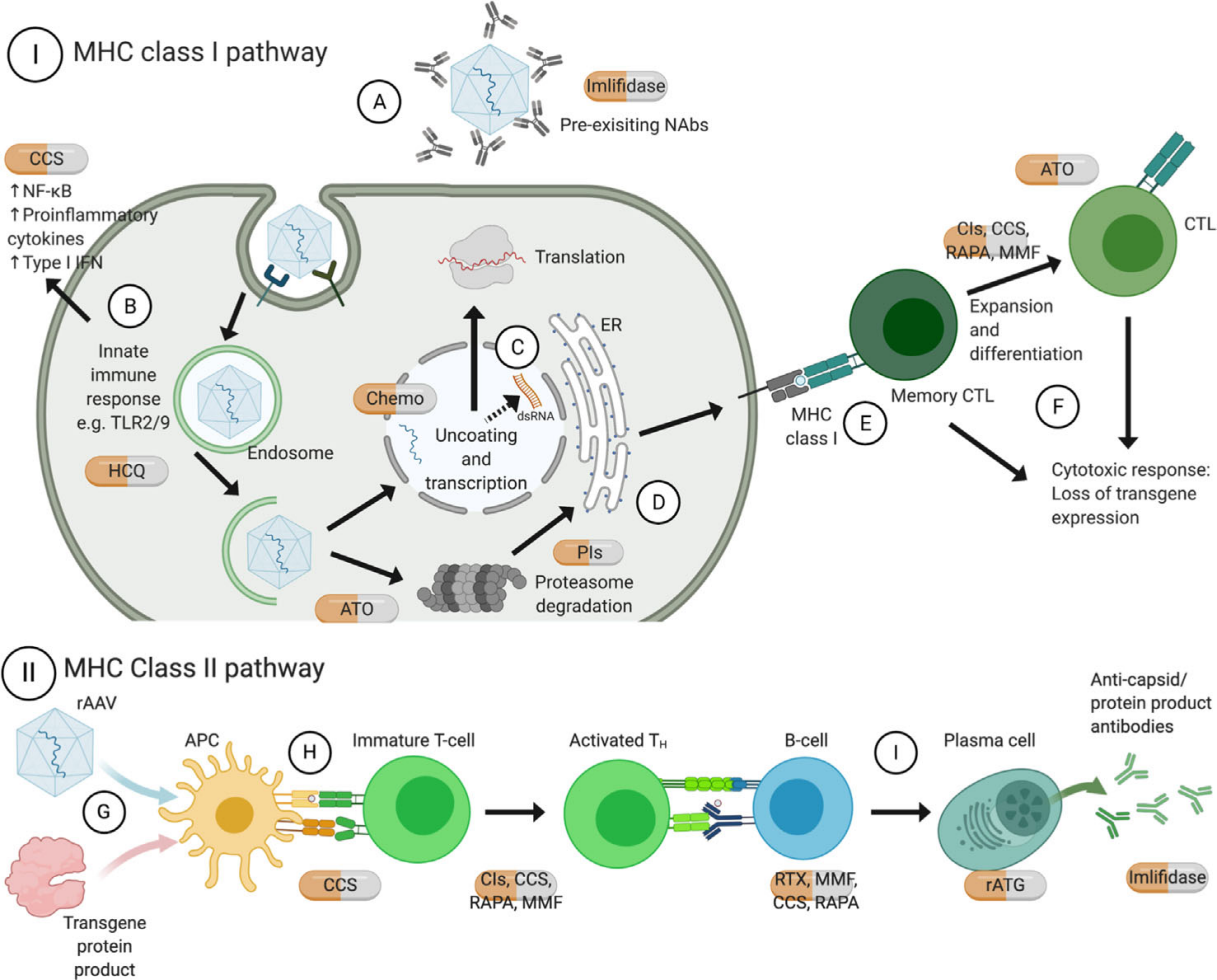
Mechanisms of action of approved pharmacotherapies for immunomodulation with rAAV gene therapy. Pre-existing NAb can inhibit receptor-mediated endocytosis thus transduction of rAAV **(A)**. TLR9 recognizes CpG motifs, and TLR2 on cell surface or endosomal membrane recognizes vector capsid, both of which lead to release of pro-inflammatory cytokines **(B)**. Recent evidence shows that ITRs facilitate bidirectional transcription to form dsRNA, which triggers cytosolic MDA5 and downstream type I interferon response **(C)**. Upon endosomal escape, rAAV can be degraded by proteasome and loaded on MHC class I by the endoplasmic reticulum **(D)**. Recognition by memory CTL **(E)** leads to expansion and differentiation into CTL, and both can commence effector functions leading to loss of transgene expression **(F)**. On the other hand, rAAV can also transduce APC, for instance dendritic cells, and transgene protein product can be phagocytosed **(G)**. They are processed by proteasomes and endosomes respectively and the antigens can be presented on MHC class II molecules **(H)**, leading to downstream activation of T_H_ and B-cells; among other actions, B cells would differentiate into plasma cells and produce antigen-specific antibodies **(I)**. Created with BioRender.com. APC, antigen presenting cells; ATO, arsenic trioxide; CCS, corticosteroids; Chemo, chemotherapeutics; CIs, calcineurin inhibitors; CTL, cytotoxic T lymphocytes; dsRNA, double-stranded ribonucleic acid; HCQ, hydroxychloroquine; IFN, interferon; IL, interleukin; ITR, inverted terminal repeats; MHC, major histocompatibility complex; MMF, mycophenolate mofetil; NAbs, neutralizing antibodies; NF-κB, nuclear factor kappa B; PIs, proteasome inhibitors; RAPA, rapamycin; rATG, rabbit anti-thymocyte globulin; RTX, rituximab; TH, T helper cells; TNF, tumor necrosis factor; TLR, toll-like receptor.

Different pharmacotherapies have been used to modulate immune responses in current *in vivo* rAAV studies. Here, with a particular focus on licensed agents, we discuss the pharmacology of each drug (**Figure 1**), and their applications in enabling safe and long-term expression of rAAV gene therapies (**Table 1**).

**Table 1:** Licensed pharmacotherapies used in preclinical and clinical studies as adjuvant to AAV gene therapies.

Drug	Licensed indication(s)	Significant adverse effects in humans	Example AAV serotype trialed	Type of study
Corticosteroids (23, 24)	Anti-inflammatory and immunosuppressive properties are used in most areas of medicine - Autoimmune diseases e.g. rheumatoid arthritis, systemic lupus erythematous (SLE) - Systemic and local inflammation - Acute exacerbation of asthma and inflammatory bowel disease	Short term treatment: adrenal suppression, hyperglycemia Long term treatment: osteoporotic fracture, insulin resistance, Cushingoid features, cataracts/glaucoma, neuropsychiatric disturbances, cardiovascular risks, muscle and skin atrophy In children: growth suppression, Cushing’s syndrome, medication-induced diabetes	AAV2 (25), scAAV9 (26)	Approved
			AAV2 (27), AAV5 (28), AAVrh10 (29), AAV-Spark100 (30), scAAV2/8 (13), scAAV5 (31)	Clinical
			AAV1 (32)	Clinical as combination
			AAVrh74 (33)	Preclinical
Rapamycin (34, 35)	Prophylaxis of organ rejection after transplantation	Thrombocytopenia, dyslipidemia, mucositis, impaired wound healing, proteinuria	AAV1 (36), AAV8 (37), AAV9 (38), AAVrh10 (29)	Clinical as combination
			AAV8 (39)	Preclinical
			AAV2 (40), (41), AAV9 (42)	Preclinical as combination
Mycophenolate mofetil (43, 44)	Prophylaxis of organ rejection after transplantation	Gastrointestinal toxicity (requiring dose reduction/discontinuation in 40-50% transplant patients), myelosuppression, infection, genotoxic	AAV8 (6), AAV2 (40) (41)	Preclinical as combination
Calcineurin inhibitors (45, 46)	Prophylaxis of organ rejection after transplantation	Narrow therapeutic index - nephrotoxicity, neurotoxicity, infection, gastrointestinal toxicity, malignancy	AAV1 (32)	Clinical as combination
			AAV8, AAV9 (47)	Preclinical
			AAV8 (48)	Preclinical as combination
Rituximab (49)	Rheumatoid arthritis, Non-Hodgkin’s lymphoma	Infusion reaction including cytokine release syndrome, infection, febrile neutropenia, myelosuppression, cardiotoxicity	AAV2 and 5 NAb (50)	*Ex vivo* human serum
			AAV1 (36), AAV9 (38), AAVrh10 (29)	Clinical as combination
			AAV8, AAV6 (51); AAV9 (42)	Preclinical as combination
Imlifidase (52)	Pre-transplant desensitization in highly sensitized, crossmatch positive renal transplant patients	Infection (pneumonia, sepsis), infusion site reaction, hepatic dysfunction, headache	AAV8, AAV-LK03 (53)	Preclinical
Proteasome inhibitors (54, 55)	Multiple myeloma	Peripheral neuropathy, myelosuppression (especially thrombocytopenia), cardiovascular events, herpes reactivation	AAV2 (56), AAV8 (57)	Preclinical
Arsenic trioxide (58)	Acute promyelocytic leukemia	Hyperleukocytosis, gastrointestinal toxicity, skin lesions, hepatic dysfunction	AAV8 (59)	Preclinical
Hydroxychloroquine (60)	Rheumatoid arthritis, SLE	Gastrointestinal effects, retinopathy, myopathy, QT prolongation (at high dosage)	AAV2 (61)	Preclinical
Rabbit anti-thymocyte globulin (62)	Prophylaxis of graft-versus-host disease or organ rejection after transplantation	Infusion reaction including cytokine release syndrome, opportunistic infection/reactivation	AAV2 (41)	Preclinical as combination

Indications, adverse effects observed in recommended dosages, and example of AAV studies are listed below. MnTBAP and Teniposide are excluded as they are not or no longer licensed in Europe and the US.

## Immunomodulation to Facilitate rAAV Gene Therapy Delivery

### Global Effects

#### Corticosteroids

Corticosteroids (CCS; methylprednisolone, prednisolone and prodrug prednisone) bind to glucocorticoid receptors modifying diverse downstream transcriptional signaling. This includes annex I, MAPK phosphatase 1, and NF-κB resulting in anti-inflammatory and immunosuppressive properties (63). They have broad inhibitory effects on both innate and adaptive immune cells by reducing pro-inflammatory cytokine and chemokines, T- and to a lesser extent, B-cells production (64). CCS are used short-term in conjunction with systemically delivered gene therapies to negate transaminitis and associated CTL-induced injury transgene loss (30, 65), and reduce T-cell infiltrates in muscular fibers in non-human primates (NHP) (33). They are also adopted in approved gene therapies for inherited retinal dystrophy (25) and spinal muscle atrophy (SMA) (26).

Subsequently increasing doses of systemic rAAV have been delivered in preclinical and clinical studies with significant hepatic sequelae. High dose intravenous AAV9 (2×10^14^ vector genomes (vg)/kg) in NHP resulted in marked transaminitis and acute liver failure (66), posing concerns over dosage related hepatotoxicity (67). Furthermore, clinical phase II trial for X-linked myotubular myopathy delivered intravenous rAAV8.AT132 (NCT03199469) 3×10^14^vg/kg in high dosage group, with 16-weeks of prednisolone commencing 1 day prior to dosing. Three patients with pre-existing intrahepatic cholestasis (68) experienced severe hepatobiliary complications culminating in death. The exact mechanisms of the hepatotoxicity remain to be elucidated. These studies however build evidence that short-course CCS alone is likely to be insufficient to inhibit formation of capsid-reactive T cells (13) and rAAV-mediated immune response with systemic high dosages. Therefore, the addition of other immunosuppressive agents maybe beneficial. In a AAVrh10-microRNA study delivering 4.2×10^14^ vg intrathecally into two adult patients, the first developed meningoradiculitis after intrathecal infusion despite corticosteroids (IV methylprednisolone on day 0 and oral prednisone tapered over 4 weeks). In the second patient, the addition of rituximab and rapamycin to the regimen resulted in a lower increase of NAb and T-cell response (29) and these drugs are further discussed.

#### Rapamycin (Sirolimus)

Rapamycin is a macrolide immunosuppressant that binds to the same intracellular target (immunophilin) as tacrolimus; however, rapamycin/FKPB12 complex inhibits a crucial cell-cycle kinase known as mammalian target of rapamycin (mTOR). Beneficial downstream effects include Treg generation, suppressing CTL and T helper (T_H_) activation and at higher doses, B-cell proliferation and differentiation (69–71).

Rapamycin has beneficial effects on circumventing existing antibodies and studied in current hemophilia gene therapy trials. Hemophilia patients develop inhibitors (antibodies) to clotting factor replacement and another cause for exclusion in gene therapy trials. In a murine hemophilia A model, rapamycin (4mg/kg three times a week) was given in addition to B-cell depleting anti-CD20 antibodies to suppress T_H_ and Treg response suppressing inhibitor development (37). Intraperitoneal prednisolone with rapamycin was shown to inhibit B-cell activation in murine spleen and bone marrow, reducing pre-existing anti-capsid immunoglobulin G (IgG) by up to 93% after 8 weeks (72). Additionally, co-administrating AAV vectors with rapamycin encapsulated in synthetic vaccine particles (SVP[Rapa]) enabled re-dosing of AAV8 at 4 × 10^12^vg/kg in mice and NHP (39). SVP [Rapa] provided sufficient reduction of B and T cell activation in an antigen-selective manner, inhibited CTL liver infiltration, and efficiently blocked memory T cell response. Potential of intramuscular rAAV9 re-administration is currently investigated for Pompe disease (NCT02240407) (73), by attenuating T and B cell response with rapamycin and rituximab respectively. Preliminary results were successful in preventing formation of anti-capsid and anti-transgene antibodies (38), with aims to enable rAAV re-administration and maintain effectiveness in different underlying mutations.

#### Mycophenolate Mofetil

Inosine monophosphate dehydrogenase (IMPDH) is the rate-limiting enzyme for guanosine nucleotide synthesis, and type II IMPDH is upregulated in activated lymphocytes. Mycophenolate mofetil (MMF), prodrug of mycophenolic acid, preferentially inhibits type II IMPDH, suppressing T and B cells proliferation (74). In mice MMF reduced rAAV transduction efficiency by depleting guanosine triphosphate required for vector genome second strand synthesis (75), but this was not observed in higher animals. No difference in AAV8-hFIX transgene expression was observed when administered with tacrolimus in NHP (6), highlighting the difficulties of recapitulating human immune system in mouse models.

### T-Cell Specific

#### Calcineurin Inhibitors

Ciclosporin and tacrolimus are immunosuppressants that inhibit calcineurin, a key signaling phosphatase, by binding to their respective immunophilins - cyclophilin and FKBP12 (76). A major downstream effect is suppression of interleukin (IL)-2 transcription, thereby inhibiting T cells differentiation, survival, and subsequent antibody production and CTL activities *via* effector T_H_ cells. Daily systemic administration of tacrolimus (0.06mg/kg/day) has been shown to prolong rAAV8 and rAAV9 expression in NHP skeletal muscle, up to 42 weeks from 8 and 16 weeks respectively (47). No generalized toxicity was reported but T-cell and macrophages infiltrations were observed.

The first approved gene therapy in Europe, alipogene tiparvovec (Glybera), incorporated 12-week immunosuppression regimen with ciclosporin (3mg/kg/day) and MMF (2g/day) (32). In the initial regimen, 9/14 subjects showed humoral and cellular response against rAAV1 (77). Subsequent study (AMT-011-02) modified the regimen to commence ciclosporin and MMF from day -3 with additional methylprednisolone on day 0 resulting in transient cellular responses without clinical sequalae (78).

Ciclosporin and tacrolimus were found to inhibit Treg proliferation and activity *in vitro* (79), and similar effects were observed in tacrolimus-treated allograft patients *ex vivo* (80); this could be detrimental in inhibiting the development of peripheral tolerance following rAAV administration. However, preclinical delivery of ciclosporin and non-depleting CD4 receptor antibody (NDCD4) have been shown to induce antigen-specific Treg, enabling AAV intravenous re-administration after 3 months (48).

### B-Cell Specific

#### Rituximab

Rituximab (RTX) is a chimeric mouse/human monoclonal antibody targeting CD20 present in pre‐B and mature B cells except plasma cells. It depletes B cells by inducing apoptosis, antibody dependent cell-mediated cytotoxicity and complement dependent cytotoxicity, thereby limiting antibody production and epitope presentation *via* MHC class II to T_H_ cells (81).

A preclinical model for hemophilia B showed RTX with ciclosporin dampened NAb response to human FIX and capsid without affecting Treg (51). As ciclosporin inhibits T_H_ cell, this further improves B-cell inhibition profile. Variable responses have been observed in RTX’s effect on reducing pre-existing AAV NAb. A small group of patients with rheumatoid arthritis were treated with combination of methotrexate and RTX, lowering anti-AAV2 and anti-AAV5 NAb in a subset of patients with variable magnitudes (50). For AAV2, 9/28 patients showed at least a half-log reduction, and inferred individuals with NAb titer ≤1:1000 were more likely to respond to RTX but the contribution of methotrexate is unknown. Considering the supportive evidence from previous AAVrh10-microRNA with RTX (29), further study in RTX application is warranted.

#### IgG-Degrading Cysteine Proteinase

Imlifidase (Idefirix, Hansa Biopharma) is a IgG-degrading cysteine protease derived from *Streptococcus pyogenes* (IdeS), which specifically cleaves opsonizing IgG at the lower hinge region of the heavy chains, resulting in a F(ab’)_2_ and a non-functioning dimeric Fc fragment (82). It could potentially overcome a limitation of RTX and cleave existing capsid-specific IgG. Using a laboratory version of IdeS with rAAV8, significant reductions in anti-AAV8 IgG and NAb levels, with enhanced liver transduction and transgene expression and observed in passively immunized murine models and naturally immunized NHP (53). Notably, the study also explored rAAV re-administration with IdeS pre-treatment in NHPs. In the first study (n=1), no induction of anti-capsid IgG and NAb, along with lower IgM and increased transgene level was observed for 21 days after second rAAV8-hFIX administration. However, this was not replicated in a larger cohort (n=5) immunized with rAAV-LK03, that developed anti-capsid IgM and IgG. Further studies are required as the IdeS dosing regimen differed between studies, and two rAAV-LK03 vectors (expressing GAA and hFVIII) were used in the latter study.

### Other Pharmacological Agents

#### Proteasome Inhibitors

Proteasome inhibitors (PIs) are licensed for multiple myeloma. Second-generation carfilzomib is irreversible and more specifically inhibits chymotrypsin-like activity than bortezomib, the reversible first-generation inhibitor, which also inhibits lysosomal and calcium-activated cellular proteases (54, 83). After endosomal escape, rAAV particles either enter the nucleus for transgene expression, or become ubiquitylated then degraded by proteasome (84). The latter pathway results in unsuccessful transduction, and capsid-derived peptides are presented to CTL by MHC class I molecules, provoking elimination of transduced cells and loss of transgene expression (85). In addition, these inhibitors may have immunomodulatory role in suppressing dendritic cells function and downstream T-cell stimulation (86).

PIs have been investigated in preclinical models for their ability to increase rAAV availability and reduce CTL responses. Bortezomib has been shown to dose-dependently decrease cell surface MHC class I antigen presentation and inhibit CTL-mediated lysis after rAAV administration *in vitro* (87). Moreover, a single bortezomib dose given with rAAV8 dosing enhanced transgene expression by >50% for one year (compared to ~10%) in hemophilia A mice, and longer in-range clotting time for at least 10 months in hemophilia A dogs (57). Both bortezomib and carfilzomib enhance rAAV2 transduction *in vitro*, but bortezomib is more efficacious than carfilzomib *in vivo* when administered by retro-orbital injection with rAAV2 (56). Although no toxicity was found in the animal models, peripheral neuropathy and myelosuppression are adverse effects observed in humans (54). Emerging evidence showing variations in PI effectiveness across cell types and AAV serotypes (88), which warrants further study.

#### Chemotherapeutics

Second strand synthesis after capsid uncoating in nucleus is long-recognized as the rate-limiting step of rAAV transduction (89); an improvement in such efficacy could allow rAAV administration at lower dose. As traditional chemotherapeutics directly or indirectly induce DNA damage, thereby initiating DNA damage response (DDR) to repair lesions (90), it has been postulated that these repair mechanisms could increase conversion of rAAV genome into dsDNA (91), or divert DDR proteins that would otherwise impede dsDNA production (92). Several chemotherapy agents were evaluated previously (91, 93) and a high throughput screening study identified teniposide, a type II topoisomerase inhibitor pharmacologically similar to etoposide, as a potent transduction enhancer (94). Tail vein injection of rAAV2-Luc with teniposide (at doses of 1×10^11^vg and 20mg/kg respectively) resulted in bioluminescence 2-log higher 48 hours post-administration without hepatotoxicity. This difference reduced to ~1 log at 8 days post-administration (study endpoint). Further study is required to determine whether the effect is sustained, and evaluate potential long-term effects of non-tissue-selective chemotherapy.

#### Agents Affecting Oxidative Stress

Oxidizing agents, such as arsenic trioxide (ATO) (59), and antioxidants, such as manganese (III) tetrakis (4-benzoic acid) porphyrin chloride (MnTBAP) (95), have been evaluated. Intraperitoneal ATO 5μg/g/day from day -2 to 2 showed 3.9-fold increase in luciferase assay 12 days after rAAV8 retro-orbital injection, with dose-dependent increase of intracellular reactive oxygen species that inhibit vector degradation pathways (59). Intraperitoneal MnTBAP 80mg/kg/day from day 0-4 reversibly downregulated CD4 on T cells, inhibiting T cell priming and humoral responses to initial rAAV1 dosing, and allowing re-administration of rAAV1 *via* a different route 28 days later (95).

#### Anti-Malarials

Hydroxychloroquine is an anti-malarial that interferes with TLRs and cyclic GMP-AMP synthase (cGAS), dampening downstream pro-inflammatory cytokine and type I IFN production (60). A study injected hydroxychloroquine subretinally (18.75μM) with rAAV2, resulting in 5.9-fold improvement in photoreceptor transgene expression (61). However, endosomal acidification is essential for rAAV escape (84), and hydroxychloroquine increases endosomal and lysosomal pH (60), this effect may not be replicated or consistent with systemic application.

### Combination Therapy

#### Triple T-Cell Directed Therapy

This study highlights importance of pharmacotherapy choice. rAAV2-hFIX (8×10^12^vg/kg) was delivered intrahepatically to NHP alongside 2-drug regimen of MMF and rapamycin compared to 3-drug adding Daclizumab (40). The addition of daclizumab resulted in decreased CD4^+^CD25^+^FoxP3^+^ Treg and consistent formation of inhibitory antibodies to hFIX; this was not observed in the 2-drug group. Daclizumab is a humanized monoclonal antibody targeting CD25 present on interleukin-2 receptor commonly found in activated T cells and CD4^+^CD25^+^FoxP3^+^ (96). This indicates careful selection of immunosuppressive agents is necessary as Treg play a critical role in regulating immune response to rAAV products, particularly observed in liver and muscle gene transfer (97).

#### Triple T-Cell Directed Therapy: Delayed rATG

Timing of T cell immunosuppressant regimen was evaluated with liver-directed rAAV2-hFIX, at 7.5×10^12^vg/kg *via* hepatic artery in NHP (41). Rabbit anti-thymocyte globulin (rATG), a rabbit polyclonal IgG, causes T-cell and plasma cell depletion and modulation of other immune effectors (98). Used with MMF (25 mg/kg) and rapamycin (4mg/kg, then 2mg/kg), a 35-day delay in rATG administration prevented formation of anti-transgene humoral response compared to commencing immunosuppression on day 0 (41). Neither group had cellular response to capsid or transgene, and 2 of 3 NHP in the delayed rATG group did not develop anti-capsid antibodies. It is possible by postponing rATG lowers the Th17/Treg ratio, allowing peripheral tolerance to the transgene product (41).

#### B and T Cell-Directed Therapy

This intensive immunosuppressive therapy included T-cell-targeting ATG and tacrolimus, B-cell targeting rituximab, with MMF and methylprednisolone to deliver global immunosuppression (99). This 5-drug regimen with rAAV5-PBGD 1×10^13^vg/kg infusion resulted in reduced T-cell response in NHP, but did not prevent NAb emergence following regimen removal. This suggests that drug selection, initiation and duration of suppression, and role of global immunosuppression are important considerations.

## Discussion

AAV gene therapy has the potential to be durable and transformative treatment for previously incurable, life-limiting genetic diseases. However, human immune responses to the viral vector, transgene, and protein product determine the therapeutic efficacy and possibility of re-administration. Studies showed cross-reactive anti-capsid NAb present at 15 years (100), CTL and Treg infiltrates at injection site after 5 years (101); and in NHP adverse effects related to high-dosage (42, 66). With the increasing applications of systemic rAAV at higher dosages in clinical trials, further understanding of innate and adaptive immune responses to rAAV gene therapies is essential to safe and efficacious treatment.

Multiple approaches are being developed to evade the host immune response such as evaluating effects of empty capsids (102), capsid engineering guided by antigenic footprints (103), and plasmapheresis (104). The use of existing licensed medications for their immunosuppression and immunomodulation properties offers the advantages of flexibility (by allowing variations of drug combinations, dose, and duration of immunosuppressive course), accessibility, and well-documented pharmacological and safety profiles. As summarized above, a range of pharmacological agents have been used in clinical and preclinical studies, and the timing of immunomodulation, duration, and drug regimen itself have all contributed to treatment efficacy. Corticosteroids are the most commonly used agents to resolve transaminitis, however, its relationship with resolution by corticosteroids and T-cell response are not always clear as observed in a hemophilia A trial (28, 105). Also, rAAV vectors and patients’ characteristics must be thoroughly evaluated to optimize safe delivery of high-dose systemic rAAV or re-dosing.

To better design immunomodulation regimens, thorough considerations of the underlying immunological mechanisms are essential. Peripheral tolerance mediated by Treg to counteract CTL responses in hepatic AAV studies remains an important area of development (106). Reports on Treg in liver and their persistence in muscle fibers after intermuscular delivery (17) further emphasizes the need for Treg-sparing therapies. Moreover, binding (non-neutralizing) antibodies in mice seemed to have a different biodistribution profile than NAb and higher efficacy in liver transduction (107). A proposed late-phase innate response triggered by ITRs’ inherent promoter activity that generates dsRNA that activates cytosolic MDA5 sensors and releases type I interferons as demonstrated in mice xenografted with human hepatocytes (108), poses further questions as to the ideal immunosuppression regimen. Lastly, the lack of fully predictive animal models (3, 109), and possibility of alternative, non-immune-mediated toxicity such as dorsal root ganglion toxicity with AAV9 (110), continue to represent challenges in safety and efficacy evaluation.

CRISPR-Cas9 is a promising therapeutic tool that allows genetic target-specific cleavage and editing (111). The first clinical trial is currently underway for Leber’s congenital amaurosis 10 (NCT03872479), EDIT-101, consists of Staphylococcus aureus Cas9 (SaCas9) and two guide RNA packaged in AAV5 vector for subretinal redelivery. One concern is that the prevalence of anti-SaCas9 antibodies and T-cell in humans are reported to be 78% (111). Studies showed pre-existing SaCas9 immunity in mice resulted in increased CTL response leading to hepatocyte apoptosis and loss of transgene (112). Although no adaptive immune response towards SaCas9 was reported (113), the eye is a relatively immunoprivileged site, these data will not necessarily predict immune response in humans or systemic administration. By gaining a precise understanding of the immune mechanisms, drug repurposing (for instance JAK inhibitors for type I interferon signaling, anti-interleukin-6 human monoclonal antibodies), alongside with how and when to immunomodulate around rAAV dosing and required duration, will help to fully maximize gene therapy safety and efficacy.

## Author Contributions

WC researched on and prepared the draft. JN reviewed and edited the manuscript. All authors contributed to the article and approved the submitted version.

## Funding

JN received funding from UK Medical Research Council MR/K02342X/1, MR/R015325/1, Great Ormond Street Hospital Children’s Charity (V1284), the Rosetrees Trust, Robert Luff Foundation and John Black Foundation (M576).

## Conflict of Interest

JN has sponsored research agreements with AskBio Europe and Rocket Pharma.

The remaining author declares that the research was conducted in the absence of any commercial or financial relationships that could be construed as a potential conflict of interest.

## References

[1] Ogden PJ Kelsic ED Sinai S Church GM. Comprehensive AAV capsid fitness landscape reveals a viral gene andenables machine-guided design. Science (2019) 366(6469):1139–43. 10.1126/science.aaw2900 PMC7197022 31780559

[2] Naso MF Tomkowicz B Perry WL Strohl WR. Adeno-Associated Virus (AAV) as a Vector for Gene Therapy. BioDrugs (2017) 31:317–34. 10.1007/s40259-017-0234-5 PMC5548848 28669112

[3] Ronzitti G Gross DA Mingozzi F. Human Immune Responses to Adeno-Associated Virus (AAV) Vectors. Front Immunol (2020) 11:670. 10.3389/fimmu.2020.00670 32362898 PMC7181373

[4] Boutin S Monteilhet V Veron P Leborgne C Benveniste O Montus MF. Prevalence of serum IgG and neutralizing factors against adeno-associated virus (AAV) types 1, 2, 5, 6, 8, and 9 in the healthy population: Implications for gene therapy using AAV vectors. Hum Gene Ther (2010) 21:704–12. 10.1089/hum.2009.182 20095819

[5] Mingozzi F Maus MV Hui DJ Sabatino DE Murphy SL Rasko JEJ. CD8+ T-cell responses to adeno-associated virus capsid in humans. Nat Med (2007) 13:419–22. 10.1038/nm1549 17369837

[6] Jiang H Couto LB Patarroyo-White S Liu T Nagy D Vargas JA. Effects of transient immunosuppression on adenoassociated,virus-mediated, liver-directed gene transfer in rhesus macaques and implications for human genetherapy. Blood (2006) 108(10):3321–8. 10.1182/blood-2006-04-017913 PMC1895424 16868252

[7] Calcedo R Vandenberghe LH Gao G Lin J Wilson JM. Worldwide Epidemiology of Neutralizing Antibodies to Adeno-Associated Viruses. J Infect Dis (2009) 199:381–90. 10.1086/595830 PMC10826927 19133809

[8] Li C Narkbunnam N Samulski RJ Asokan A Hu G Jacobson LJ. Neutralizing antibodies against adeno-associated virus examinedprospectively in pediatric patients with hemophilia. Gene Ther (2012) 19:288–94. 10.1038/gt.2011.90 21697954

[9] Hui DJ Edmonson SC Podsakoff GM Pien GC Ivanciu L Camire RM. AAV capsid CD8+ T-cell epitopes are highly conserved across AAV serotypes. Mol Ther - Methods Clin Dev (2015) 2:15029. 10.1038/mtm.2015.29 26445723 PMC4588448

[10] Vandamme C Adjali O Mingozzi F. Unraveling the Complex Story of Immune Responses to AAV Vectors Trial After Trial. Hum Gene Ther (2017) 28:1061–74. 10.1089/hum.2017.150 PMC5649404 28835127

[11] Sack BK Herzog RW Terhorst C Markusic DM. Development of gene transfer for induction of antigen-specific tolerance. Mol Ther - Methods Clin Dev (2014) 1:14013. 10.1038/mtm.2014.13 25558460 PMC4280786

[12] Manno CS Arruda VR Pierce GF Glader B Ragni M Rasko J. Successful transduction of liver in hemophilia by AAV-Factor IX and limitations imposed by the host immune response. Nat Med (2006) 12:342–7. 10.1038/nm1358 16474400

[13] Nathwani AC Reiss UM Tuddenham EGD Rosales C Chowdary P McIntosh J. Long-Term Safety and Efficacy of Factor IX Gene Therapy in Hemophilia B. N Engl J Med (2014) 371:1994–2004. 10.1056/nejmoa1407309 25409372 PMC4278802

[14] Mendell JR Campbell K Rodino-Klapac L Sahenk Z Shilling C Lewis S. Dystrophin Immunity in Duchenne’s MuscularDystrophy. N Engl J Med (2010) 363:1429–37. 10.1056/nejmoa1000228 PMC3014106 20925545

[15] Calcedo R Somanathan S Qin Q Betts MR Rech AJ Vonderheide RH. Class I-restricted T-cell responses to a polymorphic peptide in a gene therapy clinical trial for α-1-antitrypsin deficiency. Proc Natl Acad Sci USA (2017) 114(7):1655–59. 10.1073/pnas.1617726114 PMC5320988 28137880

[16] Keeler GD Markusic DM Hoffman BE. Liver induced transgene tolerance with AAV vectors. Cell Immunol (2019). 10.1016/j.cellimm.2017.12.002 PMC5988960 29576315

[17] Mueller C Chulay JD Trapnell BC Humphries M Carey B Sandhaus RA. Human treg responses allow sustained recombinant adeno-associatedvirus-mediated transgene expression. J Clin Invest (2013) 123(12):5310–8. 10.1172/JCI70314 PMC3859421 24231351

[18] Mingozzi F High KA. Immune responses to AAV vectors: Overcoming barriers to successful gene therapy. Blood (2013) 122:23–36. 10.1182/blood-2013-01-306647 23596044 PMC3701904

[19] Martino AT Suzuki M Markusic DM Zolotukhin I Ryals RC Moghimi B. The genome of self-complementary adeno-associated viral vectorsincreases Toll-like receptor 9-dependent innate immune responses in the liver. Blood (2011) 117(24):6459–68. 10.1182/blood-2010-10-314518 PMC3123017 21474674

[20] Zhu J Huang X Yang Y. The TLR9-MyD88 pathway is critical for adaptive immune responses toadenoassociated virus gene therapy vectors in mice. J Clin Invest (2009) 119(8):2388–98. 10.1172/JCI37607 PMC2719948 19587448

[21] Konkle BA Walsh CE Escobar MA Josephson NC Young G von Drygalski A. BAX 335 hemophilia B gene therapy clinical trial results: potential impact of CpG sequences on gene expression. Blood (2021) 137:763–74. 10.1182/blood.2019004625 PMC7885820 33067633

[22] Muhuri M Maeda Y Ma H Ram S Fitzgerald KA Tai PWL. Overcoming innate immune barriers that impede AAV gene therapy vectors. J Clin Invest (2021) 131:1–14. 10.1172/JCI143780 PMC7773343 33393506

[23] Buckley L Humphrey MB. Glucocorticoid-Induced Osteoporosis. N EnglJ Med (2018) 379:2547–56. 10.1056/NEJMcp1800214 30586507

[24] Liu D Ahmet A Ward L Krishnamoorthy P Mandelcorn ED Leigh R. A practical guide to the monitoring and management of the complications of systemic corticosteroid therapy. Allergy Asthma Clin Immunol (2013) 9:30. 10.1186/1710-1492-9-30 23947590 PMC3765115

[25] Russell S Bennett J Wellman JA Chung DC Yu ZF Tillman A. Efficacy and safety of voretigene neparvovec (AAV2-hRPE65v2) in patients with RPE65-mediated inherited retinal dystrophy: a randomised, controlled, open-label, phase 3 trial. Lancet (2017) 390:849–60. 10.1016/S0140-6736(17)31868-8 PMC5726391 28712537

[26] Mendell JR Al-Zaidy S Shell R Arnold WD Rodino-Klapac LR Prior TW. Single-Dose Gene-Replacement Therapy for Spinal Muscular Atrophy. N Engl J Med (2017) 377:1713–22. 10.1056/NEJMoa1706198 29091557

[27] Bouquet C Vignal Clermont C Galy A Fitoussi S Blouin L Munk MR. Immune Response and Intraocular Inflammation in Patients with Leber Hereditary Optic Neuropathy Treated with Intravitreal Injection of Recombinant Adeno-Associated Virus 2 Carrying the ND4 Gene: A Secondary Analysis of a Phase 1/2 Clinical Trial. JAMA Ophthalmol (2019) 137:399–406. 10.1001/jamaophthalmol.2018.6902 30730541 PMC6459107

[28] Rangarajan S Walsh L Lester W Perry D Madan B Laffan M. AAV5–Factor VIII Gene Transfer in Severe Hemophilia A. N Engl J Med (2017) 377:2519–30. 10.1056/nejmoa1708483 29224506

[29] Mueller C Berry JD McKenna-Yasek DM Gernoux G Owegi MA Pothier LM. SOD1 suppression with adeno-associated virus and MicroRNA in familial ALS. N Engl J Med (2020) 383:151–8. 10.1056/NEJMoa2005056 PMC11836664 32640133

[30] George LA Sullivan SK Giermasz A Rasko JEJ Samelson-Jones BJ Ducore J. Hemophilia B Gene Therapy with a High-Specific-Activity Factor IX Variant. N Engl J Med (2017) 377:2215–27. 10.1056/NEJMoa1708538 PMC6029626 29211678

[31] Miesbach W Meijer K Coppens M Kampmann P Klamroth R Schutgens R. Gene therapy with adeno-associated virus vector 5–human factor IX in adults with hemophilia B. Blood (2018) 131:1022–31. 10.1182/blood-2017-09-804419 PMC5833265 29246900

[32] Gaudet D Stroes ES Méthot J Brisson D Tremblay K Bernelot Moens SJ. Long-term retrospective analysis of gene therapy with Alipogene Tiparvovec and its effect on lipoprotein lipase deficiency-induced pancreatitis. Hum Gene Ther (2016) 27:916–25. 10.1089/hum.2015.158 27412455

[33] Cramer ML Shao G Rodino-Klapac LR Chicoine LG Martin PT. Induction of T-Cell Infiltration and Programmed Death Ligand 2Expression by Adeno-Associated Virus in Rhesus Macaque Skeletal Muscle and Modulation byPrednisone. Hum Gene Ther (2017) 28(6):493–509. 10.1089/hum.2016.113 28345428 PMC5488353

[34] Cravedi P Ruggenenti P Remuzzi G. Sirolimus for calcineurin inhibitors in organ transplantation: Contra. Kidney Int (2010) 78:1068–74. 10.1038/ki.2010.268 20703217

[35] Verhave J Boucher A Dandavino R Collette S Senécal L Hebert MJ. The incidence, management, and evolution of rapamycin-related sideeffects in kidney transplant recipients. Clin Transplant (2014) 28:616–22. 10.1111/ctr.12361 24654608

[36] Corti M Liberati C Smith BK Lawson LA Tuna IS Conlon TJ. Safety of Intradiaphragmatic Delivery of Adeno-Associated Virus-Mediated Alpha-Glucosidase (rAAV1-CMV-hGAA) Gene Therapy in Children Affected by Pompe Disease. Hum Gene Ther Clin Dev (2017) 28:208–18. 10.1089/humc.2017.146 PMC5733674 29160099

[37] Biswas M Palaschak B Kumar SRP Rana J Markusic DM. B Cell Depletion Eliminates FVIII Memory B Cells and EnhancesAAV8-coF8 Immune Tolerance Induction When Combined With Rapamycin. Front Immunol (2020) 11:1293. 10.3389/fimmu.2020.01293 32670285 PMC7327091

[38] Byrne BJ Fuller DD Smith BK Clement N Coleman K Cleaver B. Pompe disease gene therapy: neural manifestations require consideration of CNS directed therapy. Ann Transl Med (2019) 7:290–0. 10.21037/atm.2019.05.56 PMC6642929 31392202

[39] Meliani A Boisgerault F Hardet R Marmier S Collaud F Ronzitti G. Antigen-selective modulation of AAV immunogenicity with tolerogenicrapamycin nanoparticles enables successful vector re-administration. Nat Commun (2018) 9:4098. 10.1038/s41467-018-06621-3 30291246 PMC6173722

[40] Mingozzi F Hasbrouck NC Basner-Tschakarjan E Edmonson SA Hui DJ Sabatino DE. Modulation of tolerance to the transgene product in a nonhuman primate model of AAV-mediated gene transfer to liver. Blood (2007) 110:2334–41. 10.1182/blood-2007-03-080093 PMC1988950 17609423

[41] Samelson-Jones BJ Finn JD Favaro P Wright JF Arruda VR. Timing of Intensive Immunosuppression Impacts Risk of TransgeneAntibodies after AAV Gene Therapy in Nonhuman Primates. Mol Ther - Methods Clin Dev (2020) 17:1129–38. 10.1016/j.omtm.2020.05.001 PMC7256432 32490034

[42] Hordeaux J Hinderer C Goode T Buza EL Bell P Calcedo R. Toxicology Study of Intra-Cisterna Magna Adeno-Associated Virus 9 Expressing Iduronate-2-Sulfatase in Rhesus Macaques. Mol Ther - Methods Clin Dev (2018). 10.1016/j.omtm.2018.06.004 PMC6070702 30073178

[43] Omair MA Alahmadi A Johnson SR. Safety and effectiveness of mycophenolate in systemic sclerosis. A systematic review. PloS One (2015). 10.1371/journal.pone.0124205 PMC4416724 25933090

[44] Behrend M. Adverse gastrointestinal effects of mycophenolate mofetil: Aetiology, incidence and management. Drug Saf (2001). 10.2165/00002018-200124090-00002 11522119

[45] Naesens M Kuypers DRJ Sarwal M. Calcineurin inhibitor nephrotoxicity. Clin J Am Soc Nephrol (2009). 10.2215/CJN.04800908 19218475

[46] Barbarino JM Staatz CE Venkataramanan R Klein TE Altman RB. PharmGKB summary: Cyclosporine and tacrolimuspathways. Pharmacogenet Genomics (2013) 23(10):563–85. 10.1097/FPC.0b013e328364db84 PMC4119065 23922006

[47] Ishii A Okada H Hayashita-Kinoh H Shin JH Tamaoka A Okada T. rAAV8 and rAAV9-Mediated Long-Term Muscle Transduction with Tacrolimus (FK506) in Non-Human Primates. Mol Ther - Methods Clin Dev (2020) 18:44–9. 10.1016/j.omtm.2020.05.012 PMC7298335 32577431

[48] McIntosh JH Cochrane M Cobbold S Waldmann H Nathwani SA Davidoff AM. Successful attenuation of humoral immunity to viral capsid and transgenic protein following AAV-mediated gene transfer with a non-depleting CD4 antibody and cyclosporine. Gene Ther (2012) 19:78–85. 10.1038/gt.2011.64 21716299 PMC3526978

[49] Kasi PM Tawbi HA Oddis CV Kulkarni HS. Clinical review: Serious adverse events associated with the use ofrituximab - a critical care perspective. Crit Care (2012) 16(4):231. 10.1186/cc11304 22967460 PMC3580676

[50] Mingozzi F Chen Y Edmonson SC Zhou S Thurlings RM Tak PP. Prevalence and pharmacological modulation of humoral immunity to AAV vectors in gene transfer to synovial tissue. Gene Ther (2013) 20:417–24. 10.1038/gt.2012.55 PMC3473155 22786533

[51] Mingozzi F Chen Y Murphy SL Edmonson SC Tai A Price SD. Pharmacological modulation of humoral immunity in a nonhuman primatemodel of AAV gene transfer for hemophilia B. Mol Ther (Nat Publ Group) (2012) 20(7):1410–6. 10.1038/mt.2012.84 PMC3392987 22565846

[52] Al-Salama ZT. Imlifidase: First Approval. Drugs (2020) 80:1859–64. 10.1007/s40265-020-01418-5 33058042

[53] Leborgne C Barbon E Alexander JM Hanby H Delignat S Cohen DM. IgG-cleaving endopeptidase enables in vivo gene therapy in the presence of anti-AAV neutralizing antibodies. Nat Med (2020) 26:1096–101. 10.1038/s41591-020-0911-7 32483358

[54] Merin NM Kelly KR. Clinical use of proteasome inhibitors in the treatment of multiple myeloma. Pharmaceuticals (2014) 8:1. 10.3390/ph8010001 25545164 PMC4381198

[55] Schlafer D Shah KS Panjic EH Lonial S. Safety of proteasome inhibitors for treatment of multiple myeloma. Expert Opin Drug Saf (2017) 16:167–83. 10.1080/14740338.2017.1259310 27841029

[56] Mitchell AM Samulski RJ. Mechanistic Insights into the Enhancement of Adeno-Associated Virus Transduction by Proteasome Inhibitors. J Virol (2013) 87:13035–41. 10.1128/jvi.01826-13 PMC3838122 24027330

[57] Monahan PE Lothrop CD Sun J Hirsch ML Kafri T Kantor B. Proteasome inhibitors enhance gene delivery by AAV virus vectors expressing large genomes in hemophilia mouse and dog models: A strategy for broad clinical application. Mol Ther (2010) 18:1907–16. 10.1038/mt.2010.170 PMC2990516 20700109

[58] Zhang P. On arsenic trioxide in the clinical treatment of acute promyelocytic leukemia. Leuk Res Rep (2017) 7:29–32. 10.1016/j.lrr.2017.03.001 28462082 PMC5402621

[59] Mitchell AM Li C Samulski RJ. Arsenic Trioxide Stabilizes Accumulations of Adeno-Associated Virus Virions at the Perinuclear Region, Increasing Transduction In Vitro and In Vivo. J Virol (2013) 87:4571–83. 10.1128/jvi.03443-12 PMC3624361 23408604

[60] Schrezenmeier E Dörner T. Mechanisms of action of hydroxychloroquine and chloroquine:implications for rheumatology. Nat Rev Rheumatol (2020) 16:155–66. 10.1038/s41584-020-0372-x 32034323

[61] Chandler LC Barnard AR Caddy SL Patrício MI McClements ME Fu H. Enhancement of Adeno-Associated Virus-Mediated Gene Therapy UsingHydroxychloroquine in Murine and Human Tissues. Mol Ther - Methods Clin Dev (2019) 14:77–89. 10.1016/j.omtm.2019.05.012 31309129 PMC6606965

[62] Bonifazi F Rubio MT Bacigalupo A Boelens JJ Finke J Greinix H. Rabbit ATG/ATLG in preventing graft-versus-host disease after allogeneic stem cell transplantation: consensus-based recommendations by an international expert panel. Bone Marrow Transplant (2020) 55:1093–102. 10.1038/s41409-020-0792-x PMC7269907 31969678

[63] Rhen T Cidlowski JA. Antiinflammatory Action of Glucocorticoids — New Mechanismsfor Old Drugs. N Engl J Med (2005) 353:1711–23. 10.1056/nejmra050541 16236742

[64] Coutinho AE Chapman KE. The anti-inflammatory and immunosuppressive effects ofglucocorticoids, recent developments and mechanistic insights. Mol Cell Endocrinol (2011) 335(1):2–13. 10.1016/j.mce.2010.04.005 20398732 PMC3047790

[65] Nathwani AC Tuddenham EGD Rangarajan S Rosales C McIntosh J Linch DC. Adenovirus-Associated Virus Vector–Mediated Gene Transfer in Hemophilia B. N Engl J Med (2011) 365:2357–65. 10.1056/NEJMoa1108046 PMC3265081 22149959

[66] Hinderer C Katz N Buza EL Dyer C Goode T Bell P. Severe Toxicity in Nonhuman Primates and Piglets Following High-Dose Intravenous Administration of an Adeno-Associated Virus Vector Expressing Human SMN. Hum Gene Ther (2018) 29:285–98. 10.1089/hum.2018.015 PMC5865262 29378426

[67] High-dose AAV gene therapy deaths. Nat Biotechnol (2020) 38:910. 10.1038/s41587-020-0642-9 32760031

[68] Shieh PB Bönnemann CG Müller-Felber W Blaschek A Dowling JJ Kuntz NL. Re: “Moving forward after Two Deaths in a Gene Therapy Trialof Myotubular Myopathy” by Wilson and Flotte. Hum Gene Ther (2020) 31(15–16):787. 10.1089/hum.2020.217 32777938 PMC7462017

[69] Limon JJ So L Jellbauer S Chiu H Corado J Sykes SM. MTOR kinase inhibitors promote antibody class switching via mTORC2 inhibition. Proc Natl Acad Sci USA (2014). 10.1073/pnas.1407104111 PMC4250172 25385646

[70] Delgoffe GM Kole TP Zheng Y Zarek PE Matthews KL Xiao B. The mTOR Kinase Differentially Regulates Effector and Regulatory T Cell Lineage Commitment. Immunity (2009). 10.1016/j.immuni.2009.04.014 PMC2768135 19538929

[71] Zeiser R Leveson-Gower DB Zambricki EA Kambham N Beilhack A Loh J. Differential impact of mammalian target of rapamycin inhibition on CD4 +CD25+Foxp3+ regulatory T cells compared with conventional CD4+ T cells. Blood (2008) 111(1):453–62. 10.1182/blood-2007-06-094482 PMC2200823 17967941

[72] Velazquez VM Meadows AS Pineda RJ Camboni M McCarty DM Fu H. Effective Depletion of Pre-existing Anti-AAV Antibodies Requires Broad Immune Targeting. Mol Ther - Methods Clin Dev (2017) 4:159–68. 10.1016/j.omtm.2017.01.003 PMC5363314 28345001

[73] Corti M Cleaver B Clément N Conlon TJ Faris KJ Wang G. Evaluation of Readministration of a Recombinant Adeno-AssociatedVirus Vector Expressing Acid Alpha-Glucosidase in Pompe Disease: Preclinical to ClinicalPlanning. Hum Gene Ther Clin Dev (2015) 26(3):185–93. 10.1089/humc.2015.068 PMC4606909 26390092

[74] Allison AC. Mechanisms of action of mycophenolate mofetil. Lupus (2005) 14:2–8. 10.1177/096120330501400102 15803924

[75] Montenegro-Miranda PS Bloemendaal LT Kunne C De Waart DR Bosma PJ. Mycophenolate mofetil impairs transduction of single-stranded adeno-associated viral vectors. Hum Gene Ther (2011) 22:605–12. 10.1089/hum.2010.222 21222531

[76] Azzi JR Sayegh MH Mallat SG. Calcineurin Inhibitors: 40 Years Later, Can’t LiveWithout. J Immunol (2013) 191(12):5785–91. 10.4049/jimmunol.1390055 24319282

[77] Gaudet D Méthot J Déry S Brisson D Essiembre C Tremblay G. Efficacy and long-term safety of alipogene tiparvovec (AAV1-LPLS447X) gene therapy for lipoprotein lipase deficiency: An open-label trial. Gene Ther (2013) 20:361–9. 10.1038/gt.2012.43 PMC4956470 22717743

[78] Ferreira V Petry H Salmon F. Immune Responses to AAV-Vectors, the Glybera Example from Bench to Bedside. Front Immunol (2014) 5:82. 10.3389/fimmu.2014.00082 24624131 PMC3939780

[79] Miroux C Morales O Ghazal K Othman SB De Launoit Y Pancré V. In vitro effects of cyclosporine a and tacrolimus on regulatoryt-cell proliferation and function. Transplantation (2012) 94(2):123–31. 10.1097/TP.0b013e3182590d8f 22743548

[80] Akimova T Kamath BM Goebel JW Meyers KEC Rand EB Hawkins A. Differing effects of rapamycin or calcineurin inhibitor onT-Regulatory cells in pediatric liver and kidney transplant recipients. Am J Transplant (2012) 12(12):3449–61. 10.1111/j.1600-6143.2012.04269.x PMC3513508 22994804

[81] Smith MR. Rituximab (monoclonal anti-CD20 antibody): Mechanisms of action and resistance. Oncogene (2003) 22:7359–68. 10.1038/sj.onc.1206939 14576843

[82] Lorant T Bengtsson M Eich T Eriksson B-M Winstedt L Järnum S. Safety, immunogenicity, pharmacokinetics, and efficacy of degradation of anti-HLA antibodies by IdeS (imlifidase) in chronic kidney disease patients. Am J Transplant (2018) 18:2752–62. 10.1111/ajt.14733 PMC6221156 29561066

[83] Fricker LD. Proteasome Inhibitor Drugs. Annu Rev Pharmacol Toxicol (2020) 60:457–76. 10.1146/annurev-pharmtox-010919-023603 31479618

[84] Dhungel BP Bailey CG Rasko JEJ. Journey to the Center of the Cell: Tracing the Path of AAV Transduction. Trends Mol Med (2020) 27(2):172–84. 10.1016/j.molmed.2020.09.010 33071047

[85] Wang D Tai PWL Gao G. Adeno-associated virus vector as a platform for gene therapy delivery. Nat Rev Drug Discovery (2019) 18:358–78. 10.1038/s41573-019-0012-9 PMC6927556 30710128

[86] Naujokat C Berges C Höh A Wieczorek H Fuchs D Ovens J. Proteasomal chymotrypsin-like peptidase activity is required foressential functions of human monocyte-derived dendritic cells. Immunology (2007)120(1):120–32. 10.1111/j.1365-2567.2006.02487.x PMC2265869 17083604

[87] Finn JD Hui D Downey HD Dunn D Pien GC Mingozzi F. Proteasome inhibitors decrease AAV2 capsid derived peptide epitope presentation on mhc class i following transduction. Mol Ther (2010) 18:135–42. 10.1038/mt.2009.257 PMC2839204 19904235

[88] Chaanine AH Nonnenmacher M Kohlbrenner E Jin D Kovacic JC Akar FG. Effect of bortezomib on the efficacy of AAV9.SERCA2a treatment to preserve cardiac function in a rat pressure-overload model of heart failure. Gene Ther (2014) 21:379–86. 10.1038/gt.2014.7 PMC3976435 24572786

[89] Ferrari FK Samulski T Shenk T Samulski RJ. Second-strand synthesis is a rate-limiting step for efficient transduction by recombinant adeno-associated virus vectors. J Virol (1996) 70:3227–34. 10.1128/jvi.70.5.3227-3234.1996 PMC190186 8627803

[90] Woods D Turchi JJ. Chemotherapy induced DNA damage response Convergence of drugs and pathways. Cancer Biol Ther (2013) 14:379–89. 10.4161/cbt.23761 PMC3672181 23380594

[91] Russell DW Alexander IE Miller AD. DNA synthesis and topoisomerase inhibitors increase transduction by adeno-associated virus vectors. Proc Natl Acad Sci USA (1995) 92:5719–23. 10.1073/pnas.92.12.5719 PMC41768 7777575

[92] Cervelli T Palacios JA Zentilin L Mano M Schwartz RA Weitzman MD. Processing of recombinant AAV genomes occurs in specific nuclear structures that overlap with foci of DNA-damage-response proteins. J Cell Sci (2008) 121:349–57. 10.1242/jcs.003632 18216333

[93] Fields PA Arruda VR Armstrong E Chu K Mingozzi F Hagstrom JN. Risk and prevention of anti-factor IX formation in AAV-mediated genetransfer in the context of a large deletion of F9. Mol Ther (2001) 4(3):201–10. 10.1006/mthe.2001.0441 11545610

[94] Nicolson SC Li C Hirsch ML Setola V Samulski RJ. Identification and Validation of Small Molecules That Enhance Recombinant Adeno-associated Virus Transduction following High-Throughput Screens. J Virol (2016) 90:7019–31. 10.1128/jvi.02953-15 PMC4984620 27147738

[95] Da Rocha S Bigot J Onodi F Cosette J Corre G Poupiot J. Temporary Reduction of Membrane CD4 with the Antioxidant MnTBAP Is Sufficient to Prevent Immune Responses Induced by Gene Transfer. Mol Ther - Methods Clin Dev (2019) 14:285–99. 10.1016/j.omtm.2019.06.011 PMC6718808 31497619

[96] Milo R. The efficacy and safety of daclizumab and its potential role in thetreatment of multiple sclerosis. Ther Adv Neurol Disord (2014) 7(1):7–21. 10.1177/1756285613504021 24409199 PMC3886384

[97] Biswas M Kumar SRP Terhorst C Herzog RW. Gene therapy with regulatory T cells: A beneficial alliance. Front Immunol (2018) 9:554. 10.3389/fimmu.2018.00554 29616042 PMC5868074

[98] Mohty M. Mechanisms of action of antithymocyte globulin: T-cell depletion andbeyond. Leukemia (2007) 21:1387–94. 10.1038/sj.leu.2404683 17410187

[99] Unzu C Hervás-Stubbs S Sampedro A Mauleón I Mancheño U Alfaro C. Transient and intensive pharmacological immunosuppression fails to improve AAV-based liver gene transfer in non-human primates. J Transl Med (2012) 10:122. 10.1186/1479-5876-10-122 22704060 PMC3412719

[100] George LA Ragni MV Rasko JEJ Raffini LJ Samelson-Jones BJ Ozelo M. Long-Term Follow-Up of the First in Human Intravascular Delivery ofAAV for Gene Transfer: AAV2-hFIX16 for Severe Hemophilia B. Mol Ther (2020) 28(9):2073–82. 10.1016/j.ymthe.2020.06.001 PMC7474338 32559433

[101] Mueller C Gernoux G Gruntman AM Borel F Reeves EP Calcedo R. 5 Year Expression and Neutrophil Defect Repair after Gene Therapy in Alpha-1 Antitrypsin Deficiency. Mol Ther (2017) 25:1387–94. 10.1016/j.ymthe.2017.03.029 PMC5474959 28408179

[102] Mingozzi F Anguela XM Pavani G Chen Y Davidson RJ Hui DJ. Overcoming preexisting humoral immunity to AAV using capsid decoys. Sci Transl Med (2013) 5:194ra92. 10.1126/scitranslmed.3005795 PMC4095828 23863832

[103] Tse LV Klinc KA Madigan VJ Rivera RMC Wells LF Havlik LP. Structure-guided evolution of antigenically distinctadeno-associated virus variants for immune evasion. Proc Natl Acad Sci USA (2017) 114(24):E4812–21. 10.1073/pnas.1704766114 28559317 PMC5474820

[104] Monteilhet V Saheb S Boutin S Leborgne C Veron P Montus MF. A 10 patient case report on the impact of plasmapheresis uponneutralizing factors against adeno-associated virus (AAV) types 1, 2, 6, and 8. Mol Ther (2011) 19(11):2084–91. 10.1038/mt.2011.108 PMC3222518 21629225

[105] Pasi KJ Rangarajan S Mitchell N Lester W Symington E Madan B. Multiyear Follow-up of AAV5-hFVIII-SQ Gene Therapy for Hemophilia A. N Engl J Med (2020) 382:29–40. 10.1056/NEJMoa1908490 31893514

[106] Poupiot J Costa Verdera H Hardet R Colella P Collaud F Bartolo L. Role of Regulatory T Cell and Effector T Cell Exhaustion in Liver-Mediated Transgene Tolerance in Muscle. Mol Ther - Methods Clin Dev (2019) 15:83–100. 10.1016/j.omtm.2019.08.012 31649958 PMC6804827

[107] Fitzpatrick Z Leborgne C Barbon E Masat E Ronzitti G van Wittenberghe L. Influence of Pre-existing Anti-capsid Neutralizing and Binding Antibodies on AAV Vector Transduction. Mol Ther - Methods Clin Dev (2018) 9:119–29. 10.1016/j.omtm.2018.02.003 PMC5948224 29766022

[108] Shao W Earley LF Chai Z Chen X Sun J He T. Double-stranded RNA innate immune response activation from long-term adeno-associated virus vector transduction. JCI Insight (2018) 3:1–15. 10.1172/jci.insight.120474 PMC6124417 29925692

[109] Martino AT Markusic DM. Immune Response Mechanisms against AAV Vectors in Animal Models. Mol Ther - Methods Clin Dev (2020) 17:198–208. 10.1016/j.omtm.2019.12.008 31970198 PMC6965504

[110] Hordeaux J Buza EL Jeffrey B Song C Jahan T Yuan Y. MicroRNA-mediated inhibition of transgene expression reduces dorsalroot ganglion toxicity by AAV vectors in primates. Sci Transl Med (2020) 12:569. 10.1126/scitranslmed.aba9188 33177182

[111] Charlesworth CT Deshpande PS Dever DP Camarena J Lemgart VT Cromer MK. Identification of preexisting adaptive immunity to Cas9 proteins in humans. Nat Med (2019) 25:249–54. 10.1038/s41591-018-0326-x PMC7199589 30692695

[112] Li A Tanner MR Lee CM Hurley AE De Giorgi M Jarrett KE. AAV-CRISPR Gene Editing Is Negated by Pre-existing Immunity to Cas9. Mol Ther (2020) 28:1432–41. 10.1016/j.ymthe.2020.04.017 PMC7264438 32348718

[113] Maeder ML Stefanidakis M Wilson CJ Baral R Barrera LA Bounoutas GS. Development of a gene-editing approach to restore vision loss in Leber congenital amaurosis type 10. Nat Med (2019) 25:229–33. 10.1038/s41591-018-0327-9 30664785

